# Gestational diabetes mellitus and risk of type 2 diabetes 10 years after the index pregnancy in Sri Lankan women—A community based retrospective cohort study

**DOI:** 10.1371/journal.pone.0179647

**Published:** 2017-06-23

**Authors:** Himali Herath, Rasika Herath, Rajitha Wickremasinghe

**Affiliations:** 1 Department of Nutrition, Medical Research Institute, Colombo, Sri Lanka; 2 Department of Obstetrics and Gynaecology, Faculty of Medicine, University of Kelaniya, Ragama, Sri Lanka; 3 Department of Public Health, Faculty of Medicine, University of Kelaniya, Ragama, Sri Lanka; Coventry University, UNITED KINGDOM

## Abstract

**Background:**

Women with a history of gestational diabetes mellitus (GDM) have an increased risk of type 2 diabetes mellitus (T2DM) later in life compared to women with no GDM. This study was aimed to determine the risk of developing T2DM 10 years after GDM in Sri Lankan women.

**Methods:**

A retrospective cohort study was conducted in the Colombo district, Sri Lanka. 7205 women who delivered a child in 2005 were identified through Public Health Midwives in the field. Women with antenatal records were interviewed and relevant data were extracted from medical records to identify potential participants. One hundred and nineteen women who had GDM and 240 women who did not have GDM were recruited. Current diagnosis of diabetes was based on history, relevant medical records and blood reports within the past 1 year.

**Results:**

The mean duration of follow up was 10.9 (SD = 0.35) years in the GDM group and 10.8 (SD = 0.31) years in the non-GDM group. The incidence density of diabetes in the GDM group was 56.3 per 1000 person years compared to 5.4 per 1000 person years in non GDM group giving a rate ratio of 10.42 (95% CI: 6.01–19.12). A woman having GDM in the index pregnancy was 10.6 times more likely to develop diabetes within 10 years compared to women with no GDM after controlling for other confounding variables. Delivering a child after 30 years, being treated with insulin during the pregnancy and delivering a baby weighing more than 3.5 Kg were significant predictors of development of T2DM after controlling for family history of diabetes mellitus (DM), GDM in previous pregnancies, parity and gestational age at delivery.

**Conclusions:**

Women with GDM had a 10-fold higher risk of developing T2DM during a 10-year follow up period as compared to women with no GDM after controlling for other confounding variables.

## Introduction

Gestational diabetes mellitus (GDM) is defined as “any degree of glucose intolerance with onset or first recognition during pregnancy that is not clearly overt diabetes” regardless of whether insulin is used for treatment or whether the condition persists after pregnancy [1]. GDM affects approximately 7% of all pregnancies varying between 1% and 14% depending on the population studied and the diagnostic criteria used [1]. One in four live births (24%) in South East Asia are complicated with some form of hyperglycemia in pregnancy [2] and the majority of them is due to gestational diabetes. Even though national estimates on GDM prevalence are lacking in Sri Lanka, two community based studies carried out in a semi-urban and a rural district of Sri Lanka reported a GDM prevalence of 10.3% [3] and 7.2% [4], respectively, based on WHO 1999 criteria [5].

Pregnancy is considered as a “diabetogenic state” and the adaptations in the glucose metabolism ensures that needs of the rapidly growing foetus is met while maintaining adequate maternal nutrition. There is increased insulin secretion and insulin sensitivity during early pregnancy which facilitates maternal storage of fat and glycogen. There is progressive insulin resistance starting in the mid-trimester due to production of various placental hormones that serve as insulin antagonists [6]. Consequent increases in maternal glucose, free fatty acids and amino acids provide adequate energy to the foetus. In normal pregnancy, an appropriate increase in insulin secretion takes place to overcome insulin resistance and blood glucose levels remain within the normal range. However, women with underlying pre-existing metabolic disturbances (pre-gestational insulin resistance and chronic β cell dysfunction with relative insulin secretion defect) are unable to upregulate the insulin secretion and develop the clinical picture of gestational diabetes [7,8,9].

GDM is associated with both insulin resistance and impaired insulin secretion and shares the same risk factors with Type 2 diabetes mellitus (T2DM). Prevalence of GDM closely resembles that of T2DM in a population [10]. There is evidence to show that GDM is a forerunner of type 2 diabetes in predisposed women who are faced with the metabolic challenges of pregnancy [11]. GDM seems to be a significant factor associated with increasing epidemic of type 2 diabetes among women and across generations in Asia. It is estimated that 10–31% of cases of diabetes in parous women are associated with previous GDM [12].

The diagnosis of GDM has implications for the pregnancy and also for the future health of the mother and child. Although normal glucose regulation usually returns shortly after delivery, women with prior GDM are at a higher risk of developing type 2 diabetes, metabolic syndrome and cardiovascular diseases later in life [13,14,15,16]. Women with a history of GDM have at least a 7-fold increased risk of T2DM later in life compared to women with no GDM [14]. Reported conversion rates from GDM to T2DM varies ranging from 2.6–70% within a follow up period of 6 weeks to 28 years after the index pregnancy [15]. This large variation in the subsequent development of T2DM may be due to genetic differences among populations, diagnostic criteria employed to diagnose GDM and T2DM, selection criteria and duration of follow-up. The progression to type 2 diabetes increases steeply within the first 5 years after delivery, and then plateaus after 10 years [16]. In addition to exposure to GDM, several factors such as ethnicity, family history of T2DM, multiparity, advanced maternal age, treatment with insulin, presence of anthropometric risk factors (pre-pregnancy and postpartum BMI, weight gain during pregnancy) and high blood sugar levels (fasting, 1 hour and 2 hour glucose levels on OGTT) in the index pregnancy have been identified as predictors of later development of T2DM [13,14,15,16,17,18,19,20,21,22]

There is a growing body of evidence to suggest that lifestyle and pharmacological interventions targeted at women with GDM can prevent or delay the development of T2DM [21,22,23,24,25,26]. The effect of dietary and physical exercise interventions, health education and pharmacological interventions on the progression to T2DM have been investigated in randomized controlled trials and observational studies. A recent review of 30 studies aiming to prevent or delay T2DM after GDM suggests that life style and pharmacological interventions may be effective in reducing the risk of T2DM later in life [26] Since 1 in 4 women in South East Asia has hyperglycaemia in pregnancy [2], preventive strategies targeted at women with GDM would have a significant public health impact on the current epidemic of diabetes and non communicable diseases in the region.

There is paucity of data on long term risk of progression to T2DM among South Asian women who have had a pregnancy complicated by GDM in Asia. This study was conducted to determine the risk of developing T2DM 10 years after GDM and to determine factors associated with development of T2DMin Sri Lankan women.

## Materials and methods

### Study design and population

This study was conducted in 6 Medical Officer of Health (MOH) areas in Colombo district in Sri Lanka. A MOH area is a health administrative sub division in a defined geographic area within a district. The total population in the MOH areas included in the study varied from 100,000 to 200,000. Each MOH area is sub divided into Public Health Midwife (PHM) areas, which constitute the smallest field health care delivery unit in the public health system of Sri Lanka. The population of a PHM area ranges from 3000–8000. The PHM is the front line healthcare worker providing maternal and child care services in the community. The PHM maintains a paper based record keeping system with several registers related to maternal and child health. The “Birth and Immunization Register” maintained by the PHM has details of all live births which took place in her allotted area. Children were identified from this register.

Sri Lanka does not have an electronic system for storing patient records. Therefore, tracing patient held antenatal records to verify exposure status was the only option. A feasibility study was conducted to verify the availability of patient held antenatal records 10 years after delivery; we found that records were available among more than 70% of the women surveyed.

The study was conducted in two stages. In the first stage of the study, a self-administered questionnaire to obtain information on history of hyperglycaemia, availability of antenatal records and blood sugar assessment during the index pregnancy was sent to all women who delivered a child in 2005 selected for the study. We defined occurrence of hyperglycaemia in the index pregnancy as a positive answer to the question ‘Did you have diabetes during the index pregnancy’. Given the high literacy levels among women, most women are informed whether they do have diabetes during pregnancy.

**Fig 1:**
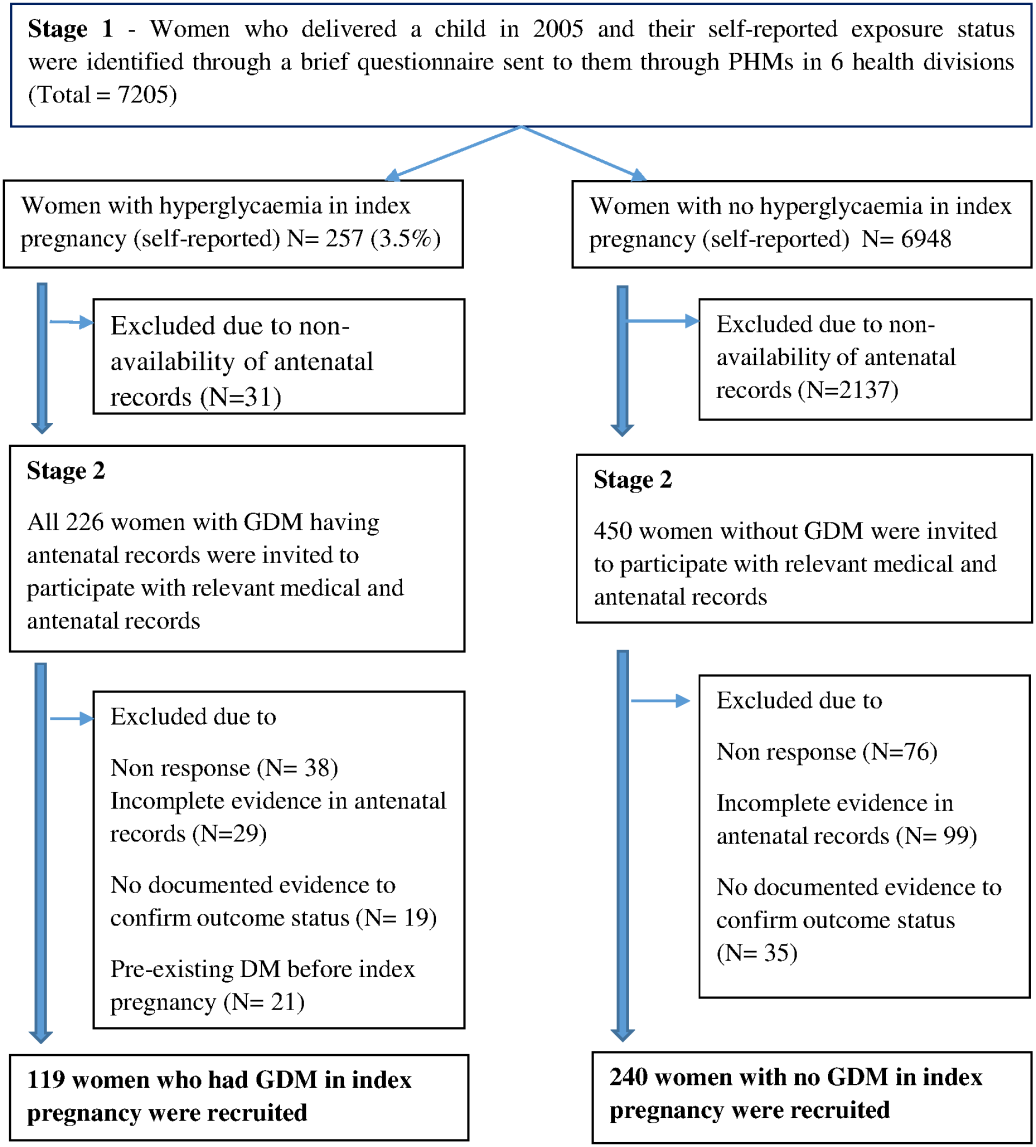
Selection of the study population.

A total of 7205 women who delivered in 2005 participated in stage 1. The prevalence of self reported hyperglyceamia in the index pregnancy was 3.5% (N = 257). Antenatal records of the index pregnancy were available in nearly 88% (N = 226) of women with hyperglycaemia in pregnancy (“exposed” group) and 69% (N = 4811) of women with no hyperglycaemia in pregnancy (“non exposed” group). All “exposed” women with antenatal records of the index pregnancy were invited to participate in the second stage of the study. For each “exposed” woman, two “non exposed” women in the same PHM area were invited to participate in the second stage. The potential participants were requested to attend the data collection session with their antenatal and medical records. The research team interviewed the potential participants and scrutinized their antenatal and medical records to identify participants meeting inclusion criteria. Women with pre-existing diabetes before the index pregnancy, inadequately documented evidence of “exposure status” (GDM vs non GDM) or “outcome status” (diabetes vs non diabetes) were excluded. 119 women who had GDM and 240 randomly selected women who did not have GDM during the index pregnancy in 2005 were recruited for the study. The detailed flow chart of participant selection is given in Fig 1.

### Data collection

Data collection was carried out by a team of doctors using an interviewer administered questionnaire with a data extraction sheet. Socio-demographic data were obtained by interviewing women.

Pregnancy related information and glycaemic status during the index pregnancy were extracted from antenatal records while outcome status (presence or absence of diabetes) was determined by patient held medical records and postpartum blood sugar reports. WHO criteria for diagnosis of diabetes in non-pregnant adults were used to determine outcome status (5).

#### Ascertainment of exposure (GDM)

Women with documentary evidence of gestational diabetes mellitus in antenatal records, glucose tolerance tests or diagnosis cards during the index pregnancy were classified as the GDM group. Diagnosis of GDM was based on WHO 1999 criteria (5) which was used in Sri Lanka in 2005.

Women with no documented evidence of GDM in antenatal records during the index pregnancy were classified as the non-GDM group. In this study, we included women with normal blood sugar assessments during the index pregnancy.

#### Ascertainment of outcome (T2DM)

Women were classified as having diabetes mellitus if they had been diagnosed by a doctor as having diabetes since the index pregnancy and currently being treated for diabetes or there was documented evidence in medical records or blood sugar reports having a FBS >126mg/dl or 75g OGTT 2 hour value ≥ 200mg/dl based on WHO criteria(5).

Women with a normal FBS or OGTT carried out within 1 year of interview were classified as non-diabetics.

### Statistical analysis

Baseline characteristics of women in the GDM and non-GDM groups were described using descriptive statistics. Variables were tested for normality using the Kolmogorov Smirnov test. Maternal age at delivery and weight at booking visit were normally distributed. Frequencies and percentages were used to summarize categorical variables and means (SD) and medians (IQR) were used to summarize continuous variables. Comparisons between GDM and non-GDM groups were done using t-tests for normally distributed variables and chi square tests for categorical variables.

The cumulative incidence, incidence density and relative risk of developing T2DM after GDM were calculated. Binary logistic regression analysis was used to explore the associations between development of diabetes and other predictors.

### Ethical considerations

The protocol was approved by the Ethics Review Committee of the Faculty of Medicine, University of Kelaniya, Sri Lanka *(Ref*. *No*.*P/24/03/2015)*. All study participants gave informed written consent. Participants with diabetes were counseled on the importance of satisfactory control of diabetes for prevention of complications of diabetes. All participants were educated about life style modification for prevention of diabetes.

## Results

### Characteristics of the study population

359 women who delivered a child in 2005 participated in the study. At the time of delivery, the age of women ranged between 13 and 46 years with a mean of 29 years. Baseline characteristics of the GDM group (n = 119) and non-GDM group (N = 240) are compared in Table 1.

**Table 1:** Characteristics of women in the GDM and non-GDM groups.

Characteristic	GDM group (N = 119)	Non GDM group (N = 240)	p value
**Index pregnancy**			
Age at delivery in years—Mean (SD)	31.7 (5.37)	27.7 (5.36)	< 0.001
Parity			
Primi	N = 40 (33.6%)	N = 121 (50.4%)	0.003
Multi	N = 79 (66.4%)	N = 119 (49.6%)	
Weight at booking visit in kg—Mean (SD)^a^	56.1(9.63)	50.5 (9.59)	< 0.001
BMI at first trimester of index pregnancy^b^			
< 18.5	N = 1 (1.5%)	N = 35 (22.3%)	< 0.001
18.5–24.9	N = 39 (57.4%)	N = 111 (70.7%)	
≥ 25	N = 28 (41.1%)	N = 11 (7%)	
GDM in previous pregnancies^c^	N = 25 (31.6%)	N = 1 (0.8%)	< 0.001
First degree relative with DM	N = 56 (47.1%)	N = 52 (21.7%)	< 0.001
Duration of pregnancy in weeks—Mean(SD)	38.3 (1.43)	39.1 (1.47)	<0.001
Gestational age at delivery ≥ 37 weeks	N = 105 (88.2%)	N = 233 (97.1%)	0.001
Delivery at a tertiary care hospital	N = 105 (88.2%)	N = 207 (86.3%)	0.6
Birth weight of index child in kg—Mean(SD)	3.1 (0.52)	2.9 (0.44)	0.001
Birth weight of index child ≥ 3.5kg	N = 31 (26.1%)	N = 22 (9.2%)	< 0.001
Sex of index child—Male	N = 48 (40.3%)	N = 112 (46.7%)	0.25
Exclusive breast feeding duration ≥ 4 months	N = 108 (90.8%)	N = 225 (93.8%)	0.30
**Sociodemographic characteristics**			
Ethnicity—Sinhala	N = 114 (95.8%)	N = 219 (91.3%)	0.39
Education level			
Primary education	N = 3 (2.5%)	N = 1 (0.4%)	0.076
Secondary education	N = 107 (89.9%)	N = 229 (95.4%)	
Tertiary education and higher	N = 9 (7.6%)	N = 10 (4.2%)	
Family Income per month			
<Rs, 50000 (< USD 340)	N = 84 (70.6%)	N = 178 (74.2%)	0.47
**Follow up**			
Current age—Mean (SD)	42.7 (5.37)	38.7 (5.36)	< 0.001
Duration of follow up in years—Mean (SD)	10.9 (0.35)	10.8 (0.31)	0.010
Developed type 2 diabetes	N = 73 (61.3%)	N = 14 (5.8%)	<0.001
Time since index pregnancy to develop diabetes (years)–Median (IQR)	3 (6.5)	8.5 (4)	0.046

^a^ Data were available in 57.1% (N = 68) of women with GDM and 65.4% (N = 157) of women without GDM

^b^ Datawere available in 57.1% (N = 68) of women with GDM and 65.4% (N = 157) of women without GDM.

^c^ Women who delivered the first child as the index child were excluded in the calculation of percentage.

Women with GDM were older, had heavier babies and had a shorter duration of pregnancy compared to non-GDM women. About half of the women in non-GDM group were in their first pregnancy compared to only one third of women with GDM (p = 0.003).

Although data were available in a sub sample of participants, women in the GDM group were heavier (p< 0.001) and had a higher BMI (p<0.001) at the first antenatal clinic visit compared to women in the non GDM group. A family history of diabetes in a first degree relative was reported in almost half of the women in the GDM group. There was no difference with regard to quality of care at delivery; the majority of women in both groups delivered at a tertiary care hospital. Ethnicity, education level and family income were not significantly different between the two groups.

### Progression to type 2 diabetes

Women with a history of GDM were older at the follow up visit. Diabetes developed in 73 (61.3%) women in the GDM group and in 14 (5.8%) women in the non GDM group during this period. The incidence density of diabetes in the GDM group was 56.3 per 1000 person years compared to 5.4 per 1000 person years in the non-GDM group giving a rate ratio of 10.42 (95% CI: 6.01–19.12).

**Fig 2:**
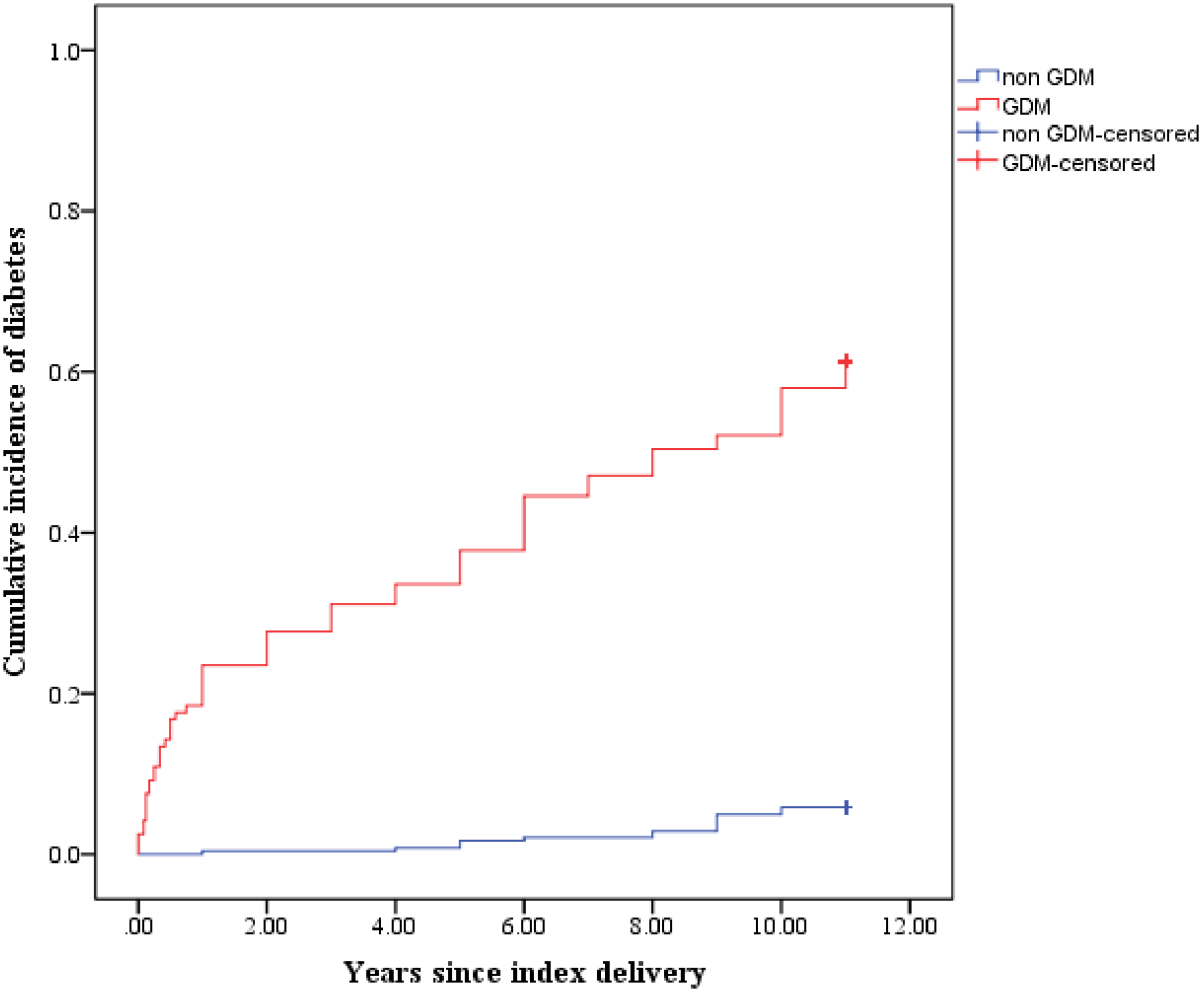
Cumulative incidence of T2DM in the GDM and non GDM groups.

The cumulative incidence of T2DM in the two groups during the follow up period is depicted in Fig 2. Time of diagnosis of T2DM was based on self-reporting by the participants with confirmed outcome status.

**Fig 3:**
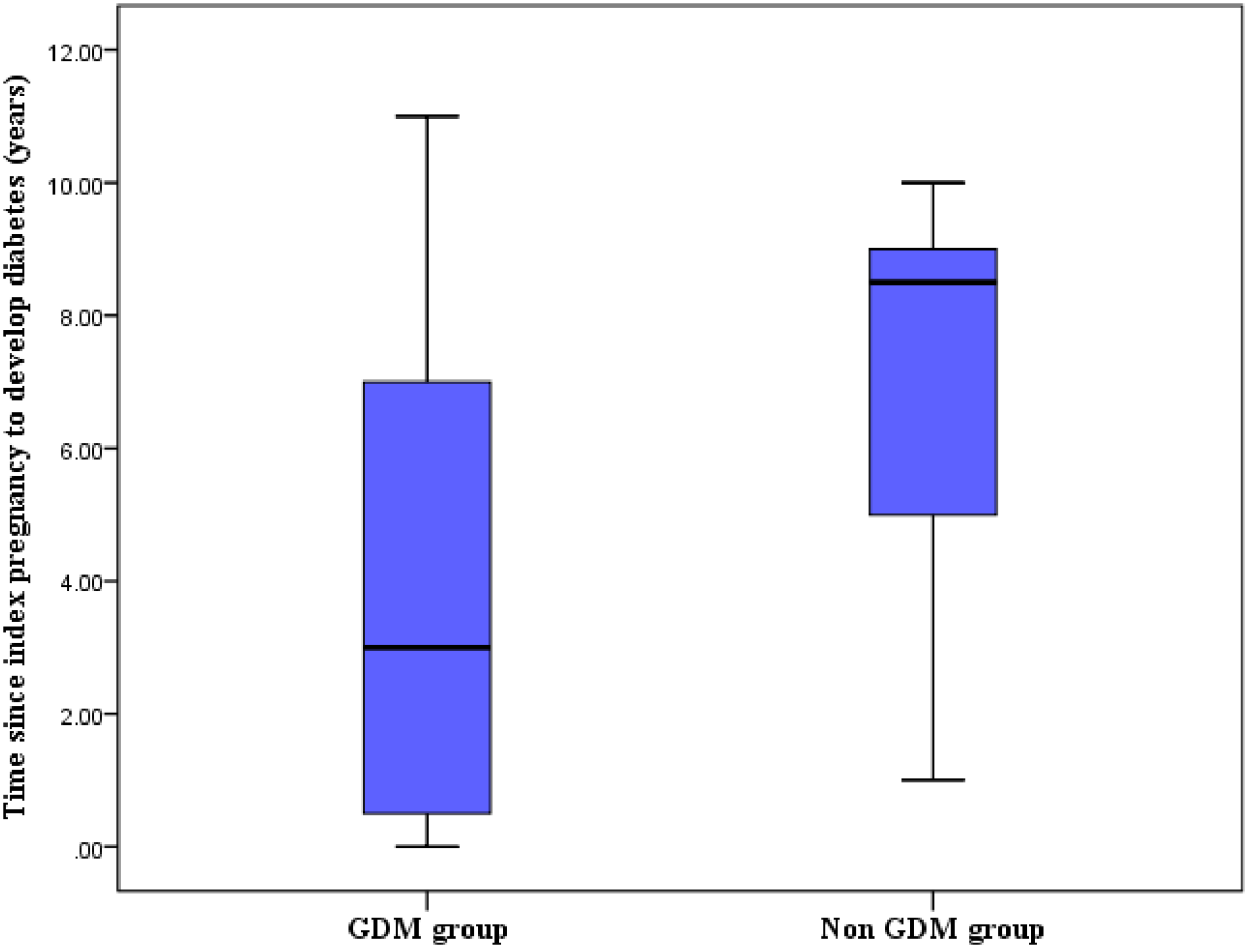
Time since index pregnancy to development of T2DM.

The median duration (IQR) for development of diabetes was 3 (6.5) years in the GDM group and 8.5 years in the non-GDM group (Fig 3).

The incidence density of diabetes by selected variables is given in Table 2.

**Table 2:** Incidence density of type 2 diabetes by selected characteristics of participants.

Characteristic		Number of women	Total person-years followed up (years)	Number of women developed T2DM postpartum	Incidence density per 1000 person-years	Rate ratio (95% CI of RR)
Age at delivery	< 25 years	81	866.7	8	9.2	1
	25–29 years	116	1252.8	18	14.3	1.55 (0.69–3.79)
	30–34 years	98	1068.2	31	29.0	3.15 (1.49–7.29)
	≥ 35 years	64	704	30	42.6	4.63 (2.18–10.74)
Educational level	Up to O/L or less	210	2268	45	19.8	1
	Up to A/L or higher	149	1609.2	42	26.1	1.32 (0.86–2.01)
Monthly family income(SLR)	< 50,000	262	2829.6	63	22.2	1
	≥ 50,000	97	1057.3	24	22.7	1.02 (0.63–1.62)
Family history of diabetes	no	251	2710.8	48	17.7	1
	yes	108	1166.4	39	33.4	1.88 (1.23–2.88)

The incidence density of diabetes increased with age; women aged ≥ 35 years were 4 times more likely to develop diabetes than women who were less than 25 years at delivery. Women with a first degree relative with diabetes were 1.88 times more likely to develop T2DM than women with no relatives with diabetes.

### Predictors of development of type 2 diabetes

Binary logistic regression analysis was performed using development of diabetes as the dependent variable and eight independent variables (GDM in index pregnancy, age at delivery, family history of T2DM in a first degree relative, history of GDM in a previous pregnancy, treatment with insulin during index pregnancy, birth weight, gestational age at delivery and parity) (Table 3). The model correctly classified 85.8% of cases. Weight at first clinic visit and BMI at first trimester were available in medical records only in a sub sample of the population (N = 68 (57%) in GDM and n = 157 (65%) in the non GDM group). Therefore, these variables were not included in the logistic regression model.

**Table 3:** Results of binary logistic regression analysis using development of diabetes as the dependent variable.

	Regression coefficient	Standard error of regression coefficient	p-value	Exp B (Odds ratio)	95% CI of Odds ratio	
					Lower	Upper
GDM in index pregnancy	2.365	0.421	<0.001	10.641	4.666	24.266
Age at delivery ≥ 30 years	0.849	0.359	0.018	2.338	1.157	4.722
Treatment with Insulin during index pregnancy	0.862	0.415	0.038	2.367	1.050	5.335
Birth weight > 3.5kg	0.880	0.421	0.037	2.410	1.056	5.50
History of GDM in previous pregnancies	0.895	0.530	0.091	2.448	0.867	6.917
Diabetes in a first degree relative at index pregnancy	0.050	0.354	0.887	1.052	0.526	2.103
Parity of index pregnancy ≥ 2	-0.257	0.367	0.484	0.774	0.377	1.589
Gestational age at delivery < 37 weeks	0.596	0.595	0.317	1.814	0.565	5.827
Constant	-3.185	0.369				

A woman having GDM in the index pregnancy was 10.6 (95% CI 4.66–24.26) times more likely to develop diabetes within 10 years after controlling for other variables as compared to women with no GDM. Delivering a child after 30 years increased the risk of developing diabetes within 10 years by 2.3 fold as compared to a younger mother. Being treated with insulin during the pregnancy increased the risk of developing diabetes within 10 years by 2.3 fold as compared to a woman not treated with insulin; likewise delivering a baby weighing more than 3.5 Kg increased the risk of developing diabetes by 2.4 fold as compared to a woman delivering a child less than 3.5 kg, after controlling for family history of DM in a first degree relative, GDM in previous pregnancies, parity and gestational age at delivery.

## Discussion

Our results show that GDM is a significant predictor of developing diabetes; women with GDM had a 10 fold higher risk of developing T2DM during a 10 year follow up period as compared to women with no GDM after controlling for other confounding variables. Our estimate is higher than the seven fold risk reported in a systematic review by Bellamy et al [14]. This highlights the importance of adherence to postpartum screening and life style modifications to prevent or delay the onset of T2DM in these women.

As described in a systematic review by Kim C et al [16], rapid conversion to diabetes is seen over the first 5 years following GDM, with a slower progression subsequently. Women in our study progressed at a more alarming rate with almost 28% of women with GDM developing T2DM within the first 2 years (Fig 2). Although the timing of diagnosis of T2DM was based on self-reporting by the women, we corroborated this information with evidence of confirmation of diagnosis. Continued surveillance of GDM women according to national guidelines on postpartum screening is imperative to detect diabetes early. Due to lack of awareness on long term risks of GDM among clinicians, healthcare workers and women, postpartum screening of women with GDM is not given priority in Sri Lanka. Moreover, due to socio-cultural practices concerning caring for the newborn child, women tend to neglect themselves thus faltering uptake of postpartum blood sugar screening facilities that are widely available in government hospitals which provide free health care services. Locally generated evidence in this study is likely to motivate clinicians and field health care workers to follow up these women more carefully to ensure postpartum screening which is recommended in the national guidelines.

We studied a cohort of Sri Lankan women who delivered a baby in 2005 to determine the risk of T2DM following GDM. The incidence of diabetes was considerably higher in women who had GDM during the index pregnancy compared to non-GDM women which is consistent with data from other countries [14–20]. Conversion rates from GDM to T2DM reported in studies vary between 2.6–70% within follow up periods of 6 weeks to 28 years after the index pregnancy [16]. Cumulative incidence of diabetes (61.3%) within 10–11 years postpartum in the current study is much higher than incidence rates reported in other countries with more than 10 years of follow up [14, 16,22,27, 28,29]. Barden et al [30] stratified women with GDM into “high risk” and “low risk” clusters based on cardio-metabolic risk measurements in pregnancy and followed them up for 10 years; type 2 diabetes was reported more frequently in the “high-risk” cluster (38.6%) compared to the “low-risk” cluster (6.2%). Still the cumulative incidence of T2DM reported in our study with similar follow up duration is much higher than in Barden et al’s “high risk” cluster.

Several studies have attempted to identify factors associated with future risk of type 2 diabetes using univariate and multivariate analyses. As reported in many studies [12, 14, 16, 23–25, 29,31,32]. having had GDM was the strongest predictor of conversion to diabetes in our study. Other factors significantly associated with development of diabetes in the present study were maternal age at delivery ≥ 30 years, birth weight of the index child >3.5kg and treatment with insulin during the index pregnancy after controlling for other variables. There was no significant association between future risk of diabetes and history of GDM in a previous pregnancy, family history of diabetes, parity, or gestational age at delivery.

In a meta analysis by Rayanagoudar et al, maternal age > 30 years and birth weight of a child >4kg were not associated with increased risk of future diabetes but insulin use during the index pregnancy was a significant predictor of developing diabetes [33]. Feig et al reported maternal age as an independent predictor of developing diabetes in a large population based study in Canada [18]. In contrast, two studies conducted in South India and Scotland did not show a statistically significant association between maternal age and development of T2DM [21, 32]

Oldfield and colleagues investigating the long term prognosis of GDM in a multiethnic population observed that insulin treatment during pregnancy was associated with future diabetes in Caucasian but not in South Asian women [22]. In contrast, women treated with insulin in our study had a two-fold higher risk of developing T2DM as compared to women not treated with insulin during the index pregnancy.

To the best of our knowledge this is the first community based study quantifying the risk of developing T2DM 10 years following GDM in Sri Lanka. Due to the unavailability of an electronic patient record system in Sri Lanka, tracking the cohort for this type of study was a tedious procedure. Even though guidelines with regard to postpartum glucose screening is available in Sri Lanka, there is no comprehensive surveillance system in the field setting to ensure postpartum glucose screening of women following GDM. These two factors were the main challenges that we faced when implementing this retrospective cohort study.

A major limitation of this study was the inability to investigate the association between maternal BMI at first trimester and risk of T2DM in the logistic regression model as data were not available for nearly 40% of the study population. BMI is calculated only if the mother is weighed in the 1^st^ trimester of pregnancy. According to the Annual Report of Family Health Bureau, the agency overseeing maternal and child health in Sri Lanka, 82.8% of pregnant women were registered by the PHM before the 12^th^ week of gestation in 2005 [34]. Hence, it is likely that some women who did not have BMI values may in fact have attended the booking visit after the 1^st^ trimester of pregnancy. Lack of a weight measurement of the mother at the booking visit did not allow us to calculate weight gain in pregnancy which is associated with GDM. However, use of birth weight would have adjusted for this potential confounding. Despite this limitation the logistic regression model we used correctly classified 86 percent of cases. Sex of the index child, breast feeding duration and ethnicity were not assessed in the logistic regression model as their distributions were not significantly different between the two groups. Other limitations of this study include not carrying out anthropometric and biochemical assessments to assess current (outcome) status.

It would have been ideal to adjust for glycaemic control and treatment of GDM women during the pregnancy. However, this information was not available. In general, all women diagnosed to have GDM are advised on dietary management and physical exercise. Those women who cannot obtain satisfactory glycaemic control with diet control and increased physical activity are started on pharmacological management. Self-monitoring of Blood Glucose Levels (BGLs) at home by pregnant women is not common in Sri Lanka. But women with GDM are seen at antenatal clinics frequently (once in 2 weeks to weekly) and a blood sugar series (fasting, pre meal and 1 hour or 2 hour post meal BGLs) is carried out at outpatient clinics to monitor glycaemic control.

The diagnosis of exposure status (“GDM”or “non-GDM”) of our sample of 359 women was confirmed for each one ensuring high reliability of our data. Detailed information was collected from patient held medical records (antenatal records, physician notes and blood investigation reports) using a pre-formatted data collection tool to ascertain exposure status, thus avoiding misclassification bias. The subsequent T2DM diagnoses were also confirmed with patient held medical records. Having data for more than 10 years of follow up in both GDM and non-GDM groups is a strength of this study.

Evidence generated in this study would be an eye opener for the women with GDM and their families, field health care workers, clinicians and policy makers to implement necessary interventions to prevent or delay the onset of T2DM. Given the high prevalence of hyperglycaemia in pregnancy in South East Asia, evidence based preventive strategies targeted at women who have had GDM are likely to have a significant population health impact on current epidemic of diabetes and non communicable diseases. This study also highlights the urgent need for universal screening of all pregnant women, particularly in South Asia for identifying these high risk women.

## Supporting information

S1 Dataset GDM and long term risk of T2DM in women—Sri Lanka.sav. (SAV)
